# Decisions about College Football during Covid-19: An Ethical Analysis

**DOI:** 10.1017/jme.2023.45

**Published:** 2023

**Authors:** Christine M. Baugh, Leonard Glantz, Michelle M. Mello

**Affiliations:** 1: UNIVERSITY OF COLORADO ANSCHUTZ MEDICAL CAMPUS, AURORA, CO, USA; 2: BOSTON UNIVERSITY, BOSTON, MA, USA; 3: STANFORD UNIVERSITY, STANFORD, CA, USA

**Keywords:** American Football, Sports Ethics, Risk, Decision-Making, Institutional Ethics, Public Health

## Abstract

This manuscript uses competitive college football as a lens into the complexities of decision-making amid the Covid-19 pandemic. Pulling together what is known about the decision-makers, the decision-making processes, the social and political context, the risks and benefits, and the underlying obligations of institutions to these athletes, we conduct an ethical analysis of the decisions surrounding the 2020 fall football season. Based on this ethical analysis, we provide key recommendations to improve similar decision processes moving forward.

The advent of the Covid-19 pandemic required university leaders to make difficult decisions balancing institutional priorities and public health objectives. After abruptly closing their campuses in spring 2020, they confronted the need for planning regarding how, or even whether, to resume activities in the fall. While the most important decisions concerned educational programs and research operations, many colleges also faced difficult choices about return to play for their football teams and other athletes.

A subgroup of universities has especially competitive and profitable college football programs. This group is often collectively called the “Power 5”, referring to the five National Collegiate Athletic Association (NCAA) Division I athletic conferences to which these schools belong (Table 1). These 65 university football programs produce over half of drafted National Football League players,^1^ despite representing merely 10% of NCAA schools fielding football teams.^2^

**Table 1:** Power Five Conferences and their Member Universities*

ACC	Big Ten	Big-12	Pac-12	SEC
Boston College	Illinois	Baylor	Arizona	Alabama
Clemson	Indiana	Iowa State	Arizona State	Arkansas
Duke	Iowa	Kansas	California	Auburn
Florida State	Maryland	Kansas State	UCLA	Florida
Georgia Tech	Michigan	Oklahoma	Colorado	Georgia
Louisville	Michigan State	Oklahoma State	Oregon	Kentucky
Miami (FL)	Minnesota	TCU	Oregon State	LSU
North Carolina	Nebraska	Texas	USC	Ole Miss
NC State	Northwestern	Texas Tech	Stanford	Mississippi State
Pittsburgh	Ohio State	West Virginia	Utah	Missouri
Syracuse	Penn State		Washington	South Carolina
Virginia	Purdue		Washington State	Tennessee
Virginia Tech	Rutgers			Texas A&M
Wake Forest	Wisconsin			Vanderbilt
Notre Dame**				

Abbreviations: ACC= Atlantic Coast Conference, SEC= Southeastern Conference

* At the time this article was written

** Notre Dame was a member of the ACC for the 2020 football season, but it is now independent.

Decisions about football for the Power 5 have high stakes both financially and in terms of equity. These teams participate in televised games with tens of millions of viewers nationwide,^3^ and receive a combined annual revenue upward of $4 billion.^4^ On average, football revenue constitutes about 3.5% of the total revenue received by Power Five schools.^5^ About half of the athletes on these teams are Black;^6^ and for low-income students, athletic scholarships may provide their only means of affording the colleges in which they are enrolled.

Power 5 football during the Covid-19 pandemic provides a useful case study for examining university decision-making concerning student athletes. Balancing risks to athletes’ health against other interests is an enduring problem for universities, but aspects of the pandemic sharpened longstanding dilemmas and created new ones. These dilemmas could well resurface during future pandemics or surges of SARS-CoV-2. Universities have legal and ethical duties to their students — including their football players — to ensure a reasonably safe environment. Football, even before the pandemic, has been a challenging context in which to apply the standard of ‘reasonably safe’ in that there are inherent risks of suffering severe or debilitating injuries as a result of participation. There are also distinctively strong competing interests (reasonable safety for athletes versus significant revenues to the university), issues of distributive justice (athletes from minority demographic groups shoulder the risks while primarily white university stakeholders reap the rewards), and challenges relating to multi-stakeholder decision-making (universities, the athletic conferences to which they belong, and the NCAA are all decision-makers in this space). Covid-19 added further complexity by requiring decision-making amid uncertainty. In this article, we use the case study of Power 5 football to highlight challenges institutional leaders face in making decisions about college sports during a pandemic and ways to improve such decisions in the future.

Power 5 football during the Covid-19 pandemic provides a useful case study for examining university decision-making concerning student athletes. Balancing risks to athletes’ health against other interests is an enduring problem for universities, but aspects of the pandemic sharpened longstanding dilemmas and created new ones. These dilemmas could well resurface during future pandemics or surges of SARS-CoV-2.

## The Chronology of Decisions Affecting the Power 5 Football Season

College sports decision-makers acted early and decisively in the pandemic. On March 12, 2020,^7^ just days after establishing its Covid-19 Advisory Panel and two weeks after the first reported U.S. Covid-19 death, the NCAA canceled all remaining winter and spring sports championships, including the popular and profitable “March Madness” men’s basketball tournament.

**Figure 1:**
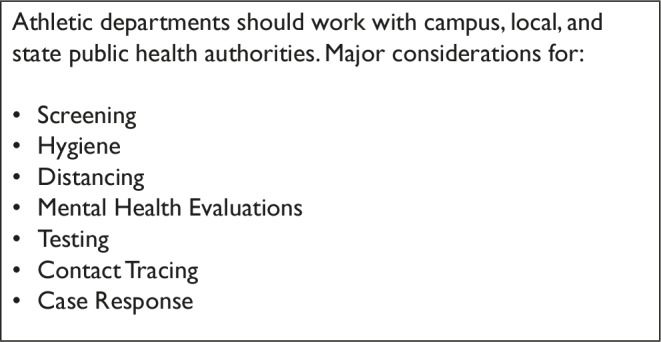
Key Points from NCAA “Resocialization of Collegiate Sport: Action Plan Considerations”

Two actions of note occurred in early summer 2020. The NCAA member schools voted to allow offseason (summer) football training on college campuses, and the NCAA released its first report on the return of college sports,^8^ setting forth considerations and best practices for institutions attempting to move forward with college athletics during fall 2020 (Figure 1).

In July 2020 several conferences announced decisions regarding fall sports. The Ivy League announced that it would postpone fall sports and its decision was quickly emulated by several other conferences. In contrast, three Power 5 conferences, the Big-Ten, Pac-12, and SEC, announced that competition would resume in the fall but be limited to other schools within their conference. Around this same time, the NCAA published a second advisory report^9^ largely updating its first, and the NCAA Football Oversight Committee approved a policy allowing greater flexibility in scheduling football competitions to account for the exigencies of team Covid-19 outbreaks.

In early August, the NCAA Board of Governors^10^ took an extraordinary action and issued a set of requirements for universities intending to proceed with fall sports competitions amid the pandemic.^11^ For the first time, the NCAA required that institutions permit athletes to opt out of participation in the athletic season without repercussions, including loss of scholarships. It also required schools to follow guidance from the NCAA and local public health authorities, established a hotline for athletes and parents to report noncompliance with health and safety protocols, and clarified that schools were required to cover Covid-19-related medical expenses for athletes, regardless of whether they contracted the disease from athletic activities. This novel policy signaled that these were, indeed, unprecedented times in college sports.

Around this same time, athletes from several conferences raised concerns about participating given the uncertainties around the virus and the unclear protections in place. SEC football players were rightfully unsatisfied when a conference official told them, “We’re going to have positive cases on every single team in the SEC. That’s a given. And we can’t prevent it.”^12^ The athletes raised concerns about the imminent return of thousands of students to campus, about participating amid the uncertainties about the health effects of the virus, and around the safety protocols in place.^13^ Pac-12 athletes threatened to boycott the season, with one player expressing concerns that “The people who are deciding whether we are going to play football are going to prioritize money over health and safety 10 times out of 10.”^14^ This was called the first collective movement by players to question why athletes are shouldering so much risk.^15^ The players demanded heightened safety protections as well as sharing of football revenue with players,^16^ but felt their concerns were dismissed in a meeting with the Pac-12 commissioner.^17^

Initial decisions about the season were announced on August 11, 2020 and were split across the conferences. The Pac-12 and Big Ten decided to postpone the football season, while the ACC, SEC, and Big-12 opted to proceed.^18^ On August 13, the NCAA canceled all Division I fall sport championships other than football (the NCAA doesn’t control this football championship),^19^ noting that fewer than 50% of schools were willing to participate in the season.^20^

The 2020 Power 5 football season was marked with inconsistencies and setbacks. The Big Ten and Pac-12 reversed their initial decisions and announced that they would hold a fall football season with a delayed start. Throughout the fall, numerous games were rescheduled, and about 1 in 5 games was canceled, because of team outbreaks or related concerns.^21^ At least ten bowl games were called off,^22^ but the end-of-season games that were held had record viewership.^23^

## Universities’ Decision-Making Processes and Outcomes in the 2020-21 Season

What explains the varied and inconstant decisions about football during the Covid-19 pandemic? Because university leaders were secretive about their decision-making, it is impossible to definitively determine the answer. In some cases, they actively took steps to hide information about the factors that influenced their decisions. For example, the Big Ten presidents made a coordinated effort to subvert state public records laws requiring release of their university emails by using a private server.^24^ Even in the absence of such overt measures to maintain secrecy, there was very limited transparency about the factors influencing decisions.

Each conference claimed that their main priority was health and safety. In explaining the initial decision not to play, the Big-12 commissioner said “there was too much uncertainty regarding potential medical risks”^25^ and the Pac-12 commissioner noted that “Unlike professional sports, college sports cannot operate in a bubble. Our athletic programs are part of broader campuses in communities where in many cases the prevalence of Covid-19 is significant.”^26^ Improved access to diagnostic testing appears to have been influential when these conferences reversed their initial decisions not to play.^27^ In some cases, however, other considerations seemed to influence decisions to play. For example, the ACC said they were following “the universities’ academic missions;” the Big Ten task force subcommittees included medical considerations, football schedule, and TV coverage. The Big Ten commissioner said simply, “The biggest argument [for proceeding with the season] is nobody’s told us that it’s poorly advised to go forward and do what we are doing.”^28^ Beyond this limited information, little is known about the factors involved in these decisions, or how leaders balanced the risks and benefits to the athletes and to the institutions.

There were varying levels of transparency regarding who was involved in the decision-making processes. Each of the Power 5 conferences created an advisory committee to help guide decision-making, policies and procedures. The exact role of the advisory committees, including their authority over the ultimate decision, was not specified. None of the conferences included student-athlete involvement, though some claimed the decisions were in line with athletes’ wishes. Based on publicly available information, committees varied significantly in their membership. Although all conferences included significant representation from school athletics departments, representation of stakeholders outside of athletics was more varied. Ultimately, the decisions were made by conference-level votes of university presidents.

In addition to the opacity regarding decision-making, there was also lack of transparency about the health outcomes of their decisions. For example, no uniform standards were imposed on football teams to disclose Covid-19 cases publicly or to the NCAA once the season had begun, and when queried by the *New York Times*, about one-third of Power 5 schools refused to provide complete information about the number of cases in their football programs.^29^ As a result, the total number of Power 5 football players, coaches, and staff who contracted Covid-19 from football is not publicly known,^30^ but a reasonable estimate is that 773 players (10.2% of all Power 5 football players) and 86 coaches or staff may have contracted the virus during the 2020-21 season (Appendix 1). While some university leaders were forthcoming in disclosing Covid-19 case counts,^31^ others purposely withheld them on the basis that disclosure would put them at a competitive disadvantage.^32^

Numerous criticisms have been made of decisions to move forward with the Power 5 football season, many by the players. The UCLA football team complained that from “neglected and mismanaged injury cases, to a now mismanaged Covid-19 pandemic, our voices have been continuously muffled.”^33^ A Michigan State offensive lineman expressed frustration that money was the main reason the season was proceeding when “guys are testing positive across the country left and right,”^34^ and another Power 5 player wrote that “the priority of coaches and administration has been set on protecting the program, not student athletes.”^35^ Some sport scholars, too, argued that “both football players and fans are sacrificial lambs for money-hungry universities.”^36^ While there were criticisms from President Trump, other politicians, parents, alumni, and athletes alike regarding the Big Ten and Pac-12’s initial decisions to postpone,^37^ these schools were also lauded for their (initial) caution.^38^

## Ethical Analysis

The experiences of these collegiate football players raise numerous questions. What obligations are owed to these athletes by organizations involved in deciding whether and how the season proceeds? From an ethical perspective, why were these decisions so challenging? In this section, we attempt to address these and other ethical and procedural questions about the 2020 Power 5 football season.

## Universities’ Obligations to Athletes

Universities have both legal and ethical obligations to their students to provide reasonably safe learning and living environments. Courts have classified the relationship between universities and their students as a “special relationship” giving rise to such a duty. Traditionally, universities have fulfilled this responsibility by, for example, ensuring pathways on campus were smooth, well lit, and free of obstacles; that campus housing was secure; and that there were policies prohibiting harassment or assault on campus.^39^ Universities also acted in cases where students were believed to pose a potential threat to themselves or others (e.g., due to mental illness). One aspect of a reasonably safe environment, even before the pandemic, was implementing policies to prevent the spread of dangerous communicable diseases, such as vaccination and quarantine. Because athletes confront additional health and safety risks by participating in university-sponsored sports, universities have responsibilities to ensure a reasonably safe playing and training environment for these athletes. For example, they must provide safe playing fields, appropriate protective gear, medical fitness exams, and access to sports medicine clinicians.

Recent changes in policies related to concussion exemplify universities acting on their responsibility of reasonable safety. Although concussion has been a known risk of participation in football, in the past decade, evidence of the serious long-term consequences has increased. This evolving understanding, combined with widely publicized cases of former college and professional football players suffering from serious cognitive and mental disabilities and untimely deaths, led college football programs to take action to reduce the risks of concussions. The NCAA mandated that universities have a concussion management protocol, educate athletes about concussion risk, remove athletes suspected of concussion from play, and require medical clearance for return to play. In addition, a few changes were instituted to alter particularly dangerous aspects of the game; for example, a penalty was instituted for contacting a defenseless athlete above the shoulder or with the crown of the head.^40^ The principles underlying these policies are informed participation (athlete education) and harm minimization. Although there have been numerous critiques of the handling of concussions in college football, the NCAA and universities have emerged from the “concussion crisis” with an ever more profitable football enterprise and perhaps some lessons on what risk management should look like in an inherently dangerous sport.

The concussion and Covid-19 contexts suggest that many universities interpret the duty to provide a “reasonably safe” environment for student athletes as requiring them to take risk-reducing steps that are feasible to implement but do not fundamentally change the nature of the game or jeopardize its continuity.

The pandemic created additional health risks that universities needed to mitigate to provide a reasonably safe environment for all students. Most universities curtailed in-person instruction and social gatherings, required masks, required testing, and/or reduced the number of students allowed on campus and in campus housing. Some universities that took these substantial and expensive steps, however, continued to permit football activities (e.g., practices) and competition — including many of the Power 5 universities.

Football activities exposed athletes to risks other students were protected from during the pandemic. In-person gatherings for training, group travel to attend competitions, the inability to wear masks and maintain social distancing during competition, and, in some cases, allowing fans to attend the competitions in person all elevated players’ risk. To mitigate the risk, universities adopted protocols such as regularly testing athletes and coaches for coronavirus, contact tracing when a participant tested positive, banning or reducing the number of spectators, and reducing training facility occupancy. These measures varied across, universities, however, because the NCAA did not require them.

The concussion and Covid-19 contexts suggest that many universities interpret the duty to provide a “reasonably safe” environment for student athletes as requiring them to take risk-reducing steps that are feasible to implement but do not fundamentally change the nature of the game or jeopardize its continuity.

## The NCAA and Its Obligations to Athletes

Since its inception in the early 1900s, the NCAA has functioned as an organizing and rulemaking body for intercollegiate sport. Importantly, the NCAA was founded to protect football athletes from the very substantial risks of the game as it was then played. Today, the NCAA continues to hold itself out as an organization dedicated to the health and safety of athletes,^41^ listing athlete wellbeing as one of its three priorities along with academics and fairness.^42^ By declaring itself an organization dedicated to the safety of athletes, the NCAA arguably created an organizational responsibility to act in accordance with that value.

Yet, in lawsuits brought by injured athletes, the NCAA has continuously denied that it has a legal duty to athletes to protect them.^43^ A notable example of this is found in what is known as the Arrington settlement, which resolved a class action lawsuit brought by a number of former athletes who claimed to have suffered concussions as a result of the NCAA’s breach of its duty to require schools to adopt measures to prevent and treat concussions.^44^ Although the NCAA settled the case for $70,000,000, it denied any responsibility. Interestingly, however, the NCAA agreed in the settlement to create a reporting process requiring schools to report every concussion suffered by a student athlete and its resolution and to adopt specific measures to prevent concussions.^45^ After this case, there could be no doubt that NCAA has the authority to require schools to adopt specific NCAA procedures to keep athletes “safe,” including reporting requirements.

The authority of the NCAA to mandate or prohibit particular behaviors by institutions and athletes is also manifested in other rules. For example, NCAA rules restrict the remuneration athletes may receive for playing and prohibited school from penalizing athletes who opted out of the 2020-21 season because of the pandemic. The NCAA’s requirements for pre-participation physical examinations and sickle cell testing and its cancellation of other fall sports seasons^46^ further demonstrate its authority and apparent sense of obligation to protect athletes. Despite holding the authority to make and enforce health and safety rules that affect these campuses’ football players, the NCAA did not use this authority to ensure uniform testing or reporting of Covid-19 cases across schools. As previously discussed, this resulted in varying university policies for disclosing Covid-19 cases, leaving many athletes in the dark about the severity of outbreaks on their teams.

An absence of documentation about why the NCAA acted as it did with regard to Power 5 football during the Covid-19 pandemic makes it difficult to divine the rationale for its decisions, but it appears that the organization prioritized independent, local decision-making over uniformity. Given local variations in the community prevalence of Covid-19 as well as varying local and state regulations relating to Covid-19, the preference for local decision-making about football is perhaps understandable. Yet, the NCAA could have imposed minimum uniform criteria and still allowed latitude for local decision-making based on local conditions. Furthermore, the difference in its approach to college football versus other fall sports, some of which were canceled even for Division I schools, raises questions about the justification for showing greater deference to colleges concerning football.

Having an external oversight agency such as NCAA helps ensure a level playing field when it comes to collegiate athlete health and safety policies. Setting minimum standards for reasonable measures to protect athletes against communicable disease spread is consonant with the NCAA’s stated values of athlete health and fairness, and arguably ethically obligatory. The “football exceptionalism” observed during the 2020-21 season therefore seems out of step with the NCAA’s ethical duties.

In the absence of NCAA leadership, colleges were left to navigate a number of thorny scientific and ethical problems in making decisions about college sports during the Covid-19 pandemic.

## Social, Political, and Ethical Complications in Colleges’ Decision-Making

While the decision to permit or prohibit college sports during a pandemic must be informed by the science and epidemiology of the disease, the decision whether or not to permit play is not a scientific one. Rather, it involves application of these facts to the values of the decision-makers. Here, we examine how key questions of fact and value affected decisions to play in the context of an evolving knowledge base about Covid-19 risk.

### Covid-19-Related Factors

Two factors directly related to the pandemic made decisions about a fall 2020 football season highly challenging. First, these decisions were made during a period of significant uncertainty as information about Covid-19 was evolving. At the time the decisions were made, there was reasonable understanding that SARS-CoV-2 was a respiratory virus that was transmitted person-to-person primarily through respiratory droplets. It was established that older individuals and individuals with certain pre-existing health conditions were more susceptible to severe illness and death, and that younger, healthier individuals (like college athletes) could be infected and spread the virus but were unlikely to suffer severe acute illness. There was a growing understanding that infection could result in prolonged illness, even among young people.^47^ There was increasing evidence that the burdens of Covid-19 disproportionately affected Black, Hispanic, and other non-white minority populations in the United States, from which most Power Five players were drawn.^48^ And even though deaths among youth, adolescents, and young adults were rare, Black, Hispanic, and other minority populations accounted for the vast majority of such deaths.^49^

During the summer decision-making period there was some evidence that Covid infection could cause a serious heart condition that could affect college-age individuals.^50^ Although later studies suggested that this condition was not a significant concern for elite athletes,^51^ that was not known at the time of the initial decision-making. (Even today, there are numerous uncertainties about the lingering and systemic effects of Covid-19 in younger individuals, including the prevalence, duration, and severity of “long Covid” symptoms.^52^) At the time of decision-making about football, early concerns about lingering cognitive effects of Covid-19 were beginning to arise.^53^ Further, there was uncertainty whether the virus was transmitted through smaller respiratory droplets or might be aerosolized.^54^ As the fall sports season got underway, major outbreaks on college campuses heightened concerns about transmission risk.^55^

In short, although it was clear that even with mitigation strategies many of the activities of college football would fall under higher-risk activities as defined by the CDC,^56^ key facts about both the probability and the severity of the risk to college athletes were not understood with a high level of certainty. While variations in colleges’ decisions about football may reflect different evaluations of the available evidence, they may also reflect different values, including the degree of trust in science among decision-makers and differing tolerance for different kinds of risks.

Second, public and political pressure heightened the stakes of decisions about the 2020 football season. Decisions were made in the runup to a highly contested national election, in a tumultuous political climate, with a president who had polarized the nation on a range of issues including the seriousness of the pandemic and simple preventive measures like wearing a mask. Numerous politicians publicly weighed in on the Power 5 football season,^57^ with President Trump tweeting “Play College Football!”^58^ Like many aspects of local pandemic response, the football decision became politicized.^59^ As one journalist put it, football became “the latest front in the country’s culture war.”^60^

Little is known about the extent to which political influences factored into university leaders’ decision-making, but particularly for publicly funded universities, this environment would have been difficult to ignore. Notably, the three Power 5 conferences who continued with their football seasons as planned were largely from states that leaned Republican.

Public pressures to proceed with the season were also apparent. The Pac-12 and Big Ten faced intense public scrutiny for their initial decisions to postpone their seasons, with parents of Big Ten athletes gathering at the conference headquarters to protest the decision.^61^ Officials at the University of Nebraska, a member of the Big Ten, publicly denounced the Big Ten decision to postpone the season, and sought opportunities for its team to play a football season outside the conference.^62^ In contrast, no outpouring of criticism was directed at divisions or schools that decided to play football.

One could argue that allowing decisions to be influenced by complaints from political leaders and members of the public is not ethically problematic, but rather, evidence of a democratic nation functioning as it should. However, if universities have obligations to protect the health of athletes and the community, as we argue below, such obligations must trump political demands that emanate from other motivations and that threaten health harms.

### Factors Related to Universities and College Football

A host of issues relating to the role and conduct of football programs within universities further complicated decisions about the 2020-21 season. These factors spanned financial pressures, the need to balance the interests of multiple stakeholders, the decentralized nature of decision-making, power dynamics between football programs and players, pressures on conceptions of consent and assumption of risk that undergird relationships between athletic programs and athletes, and equity issues.

First, decisions about the Power 5 football season had significant financial implications for the universities. Many universities were facing budgetary challenges due to decreased enrollment and new costs for Covid-19 mitigation measures on campus, including testing, contact tracing, and modifications to buildings. The NCAA and colleges also had lost approximately $800 million from the cancellation of the national basketball tournament months earlier.^63^ The prospect of an additional loss of the collective $4 billion that the Power 5 football season brings in annually, primarily to the colleges, only compounded these financial challenges.^64^ Such revenue loss has far-reaching implications, in part because some football and basketball revenue is used to subsidize other sports, as well as to pay coaching salaries and build and maintain athletic facilities.^65^ The importance of football revenue to universities arguably creates a conflict of interest for university decisionmakers when protecting athletes’ safety involves financial loss.

Second, universities have multiple constituencies that must be considered when major decisions are made, including current and future students (both athletes and non-athletes), staff, faculty, alumni, donors, boards of overseers, and the surrounding community. All are important to the flourishing of the university, and often they have different interests that must be balanced. To varying extents, these constituencies may actively exert pressure on university officials, which can further complicate decision-making. Different universities may explicitly or implicitly establish different prioritizations of these stakeholders’ respective interests — for reasons that may or may not comport with core values of the university.

Third, the decentralized nature of decision-making about football — in which NCAA permitted the Divisions and member schools to decide whether or not to play — meant that individual schools did not have political cover for making an unpopular decision not to play. No larger governing organization could be blamed. This circumstance meant that for some schools, uncommon moral courage would have been required to cancel the season.

Fourth, there has long been a significant power imbalance between football programs and football players. Athletes do not collectively bargain or have a collective voice — and yet, much rides on the athletes’ continued ability and willingness to play. Because a large proportion of players represent minoritized populations while decision-makers are largely white, these power dynamics replicate broader racial inequities in society. Decisions about the 2020-21 football season took place during the period of national unrest that followed the killing of George Floyd, making these decision-making dynamics especially fraught.

Fifth, the pandemic further exposed fissures in core principles generally described as governing the imbalanced relationships between athletic programs and athletes: consent and assumption of risk. As meaningful descriptors for the nature of athletic participation, these two principles were questionable even prior to Covid-19. For example, the concept of consent assumes that an agreement is freely and willingly made in the presence of material information about risks and benefits, but athletes often face a constrained choice set because they cannot switch among teams, may not fully appreciate the long-term risks of sports participation, and may face financial consequences for declining or discontinuing participation that are substantial enough to constitute coercion. The NCAA Board of Governors’ decision to require universities to honor scholarship obligations to athletes who opted not to participate during the pandemic addressed the last problem, but the other problems sharpened during the pandemic as the nature of the risks changed and greater uncertainty about those risks was introduced. As a result, 121 Power 5 football players opted out of the season,^66^ but despite the NCAA rule some faced challenges in doing so.^67^

The risks football players faced during the Covid-19 pandemic are distinctive in several respects. Unlike other risks such as concussion or broken bones, the risk of Covid-19 is extrinsic to the game of football. Further, athletes were no longer assuming risk only for themselves: they became a potential health threat to others, including household members, other students, and community members with whom they came into contact. Covid-19 may pose a higher risk to others (e.g., older family members and faculty) than it does to athletes themselves. The degree of uncertainty about the key dimensions of the risk — probability, severity, and duration — were higher for Covid-19 than for other football risks. The risk is also different in kind than the risks that football players knowingly ‘signed up for’ when initially choosing to participate on a school’s team. Players had to evaluate it from a different position than the one from which they made their initial decision to play — namely, as a member of team, with engrained loyalties, rather than an outsider. Finally, Covid-19 was likely considered by colleges to be a time-limited risk, which reduced universities’ incentives to invest in thoughtful mitigation measures versus just trying to make it through a season.

The ethos of football itself may have made these decisions more challenging. Toughness is a central aspect of the football mentality. Risk taking is a valorized behavior on the football “gridiron.” Sprains, strains, broken bones, torn ligaments, concussions, and other serious injuries are all common outcomes in a sport where contact and collision are inherent.^68^ Although the risks of Covid-19 are not inherent to the game, the routine and rewarded risk-taking behaviors common to the game and its athletes may have made athletes more willing to take on yet another health risk and university leaders more readily able to justify it. That football has continued with limited changes in the wake of previous health crises, such as newfound understanding that football-related brain trauma can lead to debilitating neurodegenerative disease, is a signal of the kinds of risks decision-makers are willing to and allow football athletes to assume. Indeed, relative to the other risks football players face, the risk of Covid-19 may have been viewed as modest. The distinctive culture of risk taking in football may also help explain why other sports were canceled while football was not.

Finally, the Power 5 football season raised equity issues along several dimensions. The unequal distribution of decision-making power across racial and ethnic groups has already been noted. Additionally, it raised issues of distributive justice. While the health risks of continuing sports competitions amid the pandemic were shouldered largely by the (predominantly Black and Hispanic) athletes and athletic staff, the benefits of the season, including hundreds of millions of dollars from television revenue, were reaped by athletic departments and universities.^69^ This was not a new concern: college athletes have long decried the NCAA’s strictures on their ability to monetize their skills and reputation as inequitable and unfair, culminating in a successful legal challenge before the Supreme Court in 2021.^70^ During the pandemic, however, the racial inequity assumed new importance given the risk that athletes could transmit infection to families and communities of color already disproportionately impacted by the pandemic.

A further equity concern related to prioritizing athletes for Covid-19testing and other scarce resources during the pandemic. During summer training and early in the fall football season, the availability of testing was severely limited in many communities in which the Power 5 teams resided. Yet, Power 5 teams tested their athletes, coaches, and staff weekly to daily throughout the season. Although this use of limited resources likely reduced the risks to all involved in college sports, it raises questions about the fair allocation of resources.

## Summary of Ethical Issues and Recommendations for the Future

**Figure 2:**
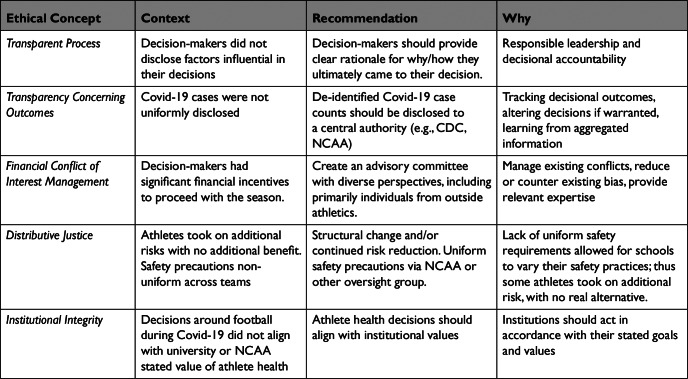
Summary of Ethical Issues and Recommendations to Improve Future Decision-Making

Here, we summarize the main ethical issues from the 2020 football season and use them as the basis for recommendations to improve complex institution-level decisions with broad health implications in the future (Figure 2). We focus on recommendations that may help institutions balance values to make thoughtful and ethical decisions.

### Transparency

Transparency is an important feature of responsible leadership and it is essential for decisional accountability. There are several applications of transparency that decision-makers could and should improve upon.

First, the decision-making process itself should be transparent. Public disclosure of who is involved, what is considered, how considerations are weighed, and why the ultimate decision was made exposes the value judgments at the heart of the decision. While deliberations may happen in private, there should be a clear, publicly available, statement justifying the ultimate decision — this should go well beyond a perfunctory statement such as ‘our highest priority was athlete health.’ Transparency in the decision-making process and decision outcome forces an honest process of considering and commenting on available evidence and in doing so may increase trust in the ultimate decision.

Additionally, there should be public disclosure of the decisional contingencies (e.g., what would make the leaders change their decision) and the anticipated decisional consequences (e.g., what are the effects of the decision on athletes, coaches, etc.). The Big Ten, for example, had a contingency that it would require a football team to pause participation if its test positivity rate and population positivity rate exceeded a certain threshold.^71^ Similarly, state Covid-19 reopening frameworks often listed criteria (e.g., case rates, hospitalization rates) that would alter opening/re-closing plans.^72^ This was a public display of rational decision-making with the primary goal of protecting public health while demonstrating an awareness of the trade-off of other socially important goods. Transparency in outcomes is essential to understanding the effects of these decisions and to allow affected individuals to continually monitor the risks they are taking on. It is also important for evaluating this and similar decisions in the future, and for allowing universities whose decisions were successful in reducing or eliminating the spread of the virus to be able to celebrate that victory.

Finally, there should be transparency between the university and the athletes about what an agreement to participate would look like under these new circumstances. Preexisting models for thinking about athlete agreement to participate in sport, including assumption of risk or (bounded) ‘consent’, tend to focus solely on the athlete side. Here, our lens includes both the university and athlete sides of the agreement. On the university side, there should be clarity around the steps the university will take to implement appropriate safety protocols, an acknolwedgement that the university will protect and uphold athletes’ decisions to cease participation, will foster continued and open communication with athletes, and will continue to support athletes as students even in the absence of athletic participation. From the athlete side, key elements of such a compact could include commitments by athletes to limit their exposures during the football season to reduce the risk of infection and transmission, follow the testing and contact tracing protocols, and submit to other requirements designed to reduce the risk of contagion to them and their school community. This kind of compact would make clear to both parties the obligations and responsibilities of the other and the specific steps that will be taken to act in accordance with their obligations. Transparent disclosure of risk should not be a cornerstone of the risk reduction approach, because transparency doesn’t reduce risks to athletes when they have limited alternative options.

### Periodic Reassessment of Decisions

Decisions should be periodically re-evaluated. Decision-making about risk under conditions of uncertainty can be improved by shortening the default time horizon for the decision to remain in place.^73^ Periodic re-evaluation of decisions effectively shortens the time horizon of the decision by providing scheduled opportunities to assess whether assumptions or facts underlying the initial decision have changed in ways that would require revisions of the initial decisions. These reassessments should be proactively scheduled and should holistically examine the facts, the decision, and the outcomes.

### Greater Use of NCAA for Uniformity

Given the NCAA’s stated priorities of safety and fairness, it could impose uniform requirements to attain these goals. Athletes at one school should not be put at greater risk than athletes at another school in the face of the same science because the NCAA has chosen to abdicate its self-declared responsibility to protect athletes. Because the pandemic affected communities differentially, it may not be desirable to centralize decisions about whether to play, but even in such circumstances, there are ways in which the NCAA can be leveraged for a shared process of collecting and analyzing relevant data. For example, the NCAA could pool epidemiologic data from schools’ experiences, which would increase collective understanding of novel viruses and specific mitigation strategies affect athletes, campuses, and perhaps surrounding communities. Here, the NCAA could provide technical support for the collection of data (e.g., common data elements) and could use the scientific expertise of their Sports Science Institute or Research arms to retain and utilize expert staff to analyze the data in ways that are important to schools.

### Managing Conflicts of Interest

The university should convene an advisory body that sits outside the football program, and ideally outside athletics altogether, which draws on expertise and perspectives from around the university. A primary goal of such a group would be to manage the biases and competing interests that some university stakeholders have in addressing the question of whether a football season should proceed. Although many universities and all Power 5 conferences did form advisory committees, the range of perspectives was sometimes limited and often centered on perspectives from within the athletics department.

This type of advisory body has several advantages. First, since it is not possible to remove the significant financial conflicts of interest or other biases that may influence the decision-making, including a range of perspectives from individuals beyond the athletics department helps attenuate these conflicts and provide alternative perspectives. Another advantage is that the committee could draw from an important range of perspectives to inform their decision-making. In selecting the committee members, leaders should consider excluding those with the most intense conflicts of interest and/or attempting to balance the biases on the committee.

This committee should represent a range of perspectives including those relevant for understanding the scientific problem (e.g., epidemiology, immunology), ethical considerations, community considerations, considerations specifically for individuals from minority backgrounds, feasibility of the implementation of a range of mitigation strategies, experience with weighing risks and benefits, financial implications, etc. Having a range of perspectives can help create a productive dialogue and balance biased perspectives (e.g., public health stakeholders may be biased toward more restriction whereas athletics stakeholders may be biased toward proceeding with the game). That individuals have these biases is not inherently problematic. When those biases drive decision-making or are unbalanced among decision-makers, problems can arise.

The authority of this body, its membership, considerations, ultimate recommendation, and rationale for that recommendation should be transparent and publicly available (e.g., posted on the school website). This transparency will help attenuate any strong biases in reasoning, and elucidate the factors, values, considerations, and priorities of the committee and the university.

### Balancing Values

While this decision should be informed by the data, it is also a decision that should be informed by values. Reasonable people looking at the same set of facts may come to different decisions depending on what values they prioritize. With limited information about the decision-making processes and criteria, we do not know what values were considered or prioritized, or even who was balancing these values and priorities. Numerous scholars have written on the challenges faced by those charged with athlete health and wellbeing.^74^ Although much of this scholarship has focused on ethical issues facing the sports medicine clinician, some of the broader lessons are relevant to our discussion. So, here we describe and contextualize some of the values that should have been relevant to the decision-making process including: individual autonomy, beneficence, nonmaleficence, institutional integrity, and justice.

Decision-makers should consider athletes’ individual autonomy. Although athletes’ autonomy is regularly limited in the case of football, decision-makers should consider whether their actions further limit athletes’ autonomy and, if so, the justification for doing so. Decisions may enhance athletes’ autonomy. For example, the decision to allow athletes to opt out of the season without repercussion represents an enhancement in autonomy — athletes cannot normally opt out of the season and maintain a scholarship. Often balanced against individual autonomy is paternalism. As in the case of not allowing an athlete to play while he has a concussion, decision-makers can set limits on individual autonomy when it is decided that the risks to the individual or others outweigh the value of allowing the individual to make a free decision. Institutions should not base their decisions on the desire of these athletes to play the game. As noted above, the decision about whether a college athlete can play in a particular game is not theirs to make; neither is the decision whether the school participates in a football season.

Decision-makers should consider how they can do good and avoid harm, the concepts of beneficence and nonmaleficence. Applying these principles in the context of the pandemic is challenging. Is it better (in terms of beneficence/nonmaleficence) to allow athletes opportunities to play, perhaps living out a lifelong dream or giving themselves the small chance to play professionally? Or is it better to avoid the potential unknown short- and/or long-term harm posed by Covid-19? Is it better for a school to honor its agreement with the athletes that they would allow them to compete in football, or to go back on these agreementst to avoid the harms that Covid-19 could have to athletes, university students, and staff, and the surrounding community? In addressing these considerations, university leaders should account for both the possible benefits and harms to the athletes, as well as to the many other university stakeholders. To balance benefits and harms, decision-makers must first identify what the benefits and harms are and how these will be distributed. Additional suggestions provided (e.g., advisory committee) may advance this process.

Decision-makers must consider and publicly explain how their decisions embody the institution’s own goals and values, sometimes called institutional integrity. For example, if the university’s primary mission is to train the leaders of the next generation, then it should consider how its decision furthers that mission. Similarly, if the NCAA’s stated goals are to promote athlete health and fairness then it should prioritize those values in its decisions and actions. There may be multiple decisions or actions that could all work to further these goals, of course.

## Conclusion

In this article we describe how decisions were made to return college football players to in-person competition at a time when other in-person college activities (and many societal activities) were sharply limited. We analyze the many pressures college administrators faced when confronted with the decisions whether to allow an elective amateur activity. Due to the lack of transparency about how these decisions were made, we infer the values and criteria the decision-makers applied. Whether there will be a similar set of sudden, dire circumstances in the future that will require similar decisions to be made is unknown. We hope not. Still, we think lessons learned from examining this novel circumstance can improve future decision-making. The primary lesson is the need for more transparency so that the decisions can be evaluated for fairness. We also recommend clarification of authority and responsibility for decision-making. We have uncovered areas in the decision-making process in elite college sports that would benefit from further exploration. We believe that our observations, findings, and suggestions can improve the decision-making process at times of great institutional stress, and where decision-makers must weigh conflicting values and goals to determine whether it is fair and just to expose college athletes to imperfectly known risks.

## Appendix 1 Estimated Cases of Covid-19 Among Power 5 Football Players, Coaches, and Staff

**Methods:** This estimate drew heavily from a database of publicly disclosed cases of Covid-19 among college athletes, coaches and staff as of Sept. 10, 2021. From this database, we counted every instance where a positive test was reported specifically for the football players, coaches, or staff. Cases that were not specific to football were excluded. If there were two articles reporting the same counts within a day or two of each other these were counted as one on the assumption that they were likely referencing the same cases. If an article said ‘multiple cases’ or ‘a couple’, this was counted as 2 cases on the basis that this would be the minimum number they could be referencing. Because of these choices and because public disclosure of cases was voluntary and uneven across colleges, the numbers presented are likely an underestimate.

**Appendix Table 1:** Estimated Covid-19 Cases Among Power 5 Football Players, Coaches, and Staff

Conference	School	Players	Coaches	Staff
ACC	Clemson	40	0	0
ACC	Louisville	10	0	5
ACC	Virginia	7	1	0
ACC	Wake Forest	4	0	0
ACC	Boston College	3	0	0
ACC	Miami (Fla.)	3	1	0
ACC	Florida State	2	1	0
ACC	Syracuse	1	0	0
ACC	Duke	0	0	0
ACC	Georgia Tech	0	0	0
ACC	NC State	0	0	0
ACC	North Carolina	0	0	0
ACC	Pittsburgh	0	0	0
ACC	Virginia Tech	0	0	0
Big 12	Texas Tech	49	1	0
Big 12	Kansas	43	1	0
Big 12	Oklahoma	33	0	0
Big 12	WVU	33	0	0
Big 12	Baylor	29	0	14
Big 12	Kansas State	20	0	0
Big 12	Texas	5	0	0
Big 12	Oklahoma State	4	0	0
Big 12	Iowa State	0	0	0
Big 12	TCU	0	0	1
Big Ten	Wisconsin	89	1	10
Big Ten	Michigan	40	0	0
Big Ten	Minnesota	36	0	39
Big Ten	Maryland	23	1	0
Big Ten	Illinois	17	0	0
Big Ten	Rutgers	17	0	0
Big Ten	Indiana	11	0	0
Big Ten	Nebraska	5	0	1
Big Ten	Ohio State	5	1	0
Big Ten	Northwestern	1	0	0
Big Ten	Iowa	0	1	0
Big Ten	Michigan State	0	0	2
Big Ten	Penn State	0	0	0
Big Ten	Purdue	0	1	0
Pac-12	UCLA	14	1	5
Pac-12	Arizona State	2	1	0
Pac-12	USC	2	0	0
Pac-12	California	1	0	0
Pac-12	Oregon State	1	0	0
Pac-12	Washington State	1	0	0
Pac-12	Arizona	0	0	0
Pac-12	Colorado	0	0	0
Pac-12	Oregon	0	0	0
Pac-12	Stanford	0	0	0
Pac-12	Washington	0	0	0
SEC	Florida	77	0	0
SEC	Auburn	58	0	3
SEC	Tennessee	34	1	0
SEC	Missouri	14	0	0
SEC	Alabama	13	1	0
SEC	Georgia	7	0	0
SEC	LSU	6	0	0
SEC	Mississippi State	5	0	0
SEC	Ole Miss	3	0	0
SEC	Vanderbilt	3	0	0
SEC	Texas A&M	2	0	0
SEC	Arkansas	0	1	0
SEC	Kentucky	0	0	0
